# Analysis of the antiparasitic and anticancer activity of the coconut palm (*Cocos nucifera* L. ARECACEAE) from the natural reserve of Punta Patiño, Darién

**DOI:** 10.1371/journal.pone.0214193

**Published:** 2019-04-02

**Authors:** Nicole M. Tayler, Cristopher A. Boya, Liuris Herrera, Jamie Moy, Michelle Ng, Laura Pineda, Alejandro Almanza, Sara Rosero, Lorena M. Coronado, Ricardo Correa, Ricardo Santamaría, Zuleima Caballero, Armando A. Durant-Archibold, Kevin J. Tidgewell, Marcy J. Balunas, William H. Gerwick, Alida Spadafora, Marcelino Gutiérrez, Carmenza Spadafora

**Affiliations:** 1 Centro de Biología Celular y Molecular de Enfermedades, Instituto de Investigaciones Científicas y Servicios de Alta Tecnología (INDICASAT AIP), City of Knowledge, Apartado, Panama, Republic of Panama; 2 Department of Biotechnology, Acharya Nagarjuna University, Guntur, A.P., India; 3 Centro de Biodiversidad y Descubrimiento de Drogas, INDICASAT AIP, City of Knowledge, Apartado, Panama, Republic of Panama; 4 Smithsonian Tropical Research Institute, Balboa, Ancon, Apartado, Panama, Republic of Panama; 5 Division of Medicinal Chemistry, Department of Pharmaceutical Sciences, University of Connecticut, Storrs, Connecticut, United States of America; 6 Skaggs School of Pharmacy and Pharmaceutical Sciences Scripps Institution of Oceanography, University of California San Diego, La Jolla, California, United States of America; 7 Center for Marine Biotechnology and Biomedicine, Scripps Institution of Oceanography, University of California San Diego, La Jolla, California, United States of America; 8 Asociación Nacional para la Conservación de la Naturaleza (ANCON), Balboa, Ancon, Apartado, Panama, Republic of Panama; University of British Columbia, CANADA

## Abstract

*Cocos nucifera* (*C*. *nucifera*) (the coconut palm tree) has been traditionally used to fight a number of human diseases, but only a few studies have tested its components against parasites such as those that cause malaria. In this study, *C*. *nucifera* samples were collected from a private natural reserve in Punta Patiño, Darien, Panama. The husk, leaves, pulp, and milk *of C*. *nucifera* were extracted and evaluated against the parasites that cause Chagas’ disease or American trypanosomiasis (*Trypanosoma cruzi*), leishmaniasis (*Leishmania donovani*) and malaria (*Plasmodium falciparum*), as well as against a line of breast cancer cells. While there was no activity in the rest of the tests, five and fifteen-minute aqueous decoctions of leaves showed antiplasmodial activity at 10% v/v concentration. Removal of some HPLC fractions resulted in loss of activity, pointing to the presence of synergy between the components of the decoction. Chemical molecules were separated and identified using an ultra-performance liquid chromatography (UPLC) approach coupled to tandem mass spectrometry (LC–MS/MS) using atmospheric pressure chemical ionization quadrupole–time of flight mass spectrometry (APCI–Q–TOF–MS) and molecular networking analysis, revealing the presence of compounds including polyphenol, flavone, sterol, fatty acid and chlorophyll families, among others.

## Introduction

In recent decades, the search for biologically active compounds in nature has intensified, following the failure of commercially available drugs due to increasing resistance in the parasites and high costs of production. Thus, the search for natural products is taking renewed efforts to discover active molecules from different sources of biodiversity.

Panama is home to one of the richest and most unique biodiversity regions of the planet. The Mesoamerica hotspot, which includes the wet and moist forests of Panama's Darién Province, is rich in thousands of species of plants, animals and microorganisms[1]. A large private natural reserve located in Punta Patiño in the Province of Darién (Patiño Nature Reserve, Chepigana district, Darien region. UTM: 801032 E 913730 N Zone 17P) is under the stewardship of the National Association for the Conservation of Nature (ANCON), a non-government organization, and encompasses large extensions of coconut palm plantations (*C*. *nucifera*) belonging to the *Arecaceae* family. It has traditionally been used in countries of South Asia, Africa, and America, for treating arthritis, diarrhea, fevers, inflammation, skin infections, miscarriage prevention, asthma, female sterility and as a diuretic, among others[2]. A leaf extract from the coconut plant decreases amyloid-β_1–42_ aggregation and paralysis[3]. Catechins and epicatechins were responsible for the antiparasitic activity from the husk of *Cocos nucifera* against *Leishmania* parasites[4]. In another study, husk fibers from *C*. *nucifera* containing alkaloids, tannins, and flavonoids were active against the *P*. *falciparum* W2 strain. The same extract fraction was active *in vivo* against *P*. *berghei* NK65, causing more than 50% reduction in parasitemia on days 4 and 6 after inoculation at various doses administered[5].

An estimated 17% of all infectious diseases in humans come from vector borne parasites. *P*. *falciparum*, the causative agent of the most devastating form of malaria, is the principal parasitic disease worldwide. Malaria alone causes 212 million new cases globally[6]. On their part, all trypanosomatids, which includes *Leishmania* parasites, take a high toll on public health: approximately 700,000 to 1 million new cases and 20,000 to 30,000 deaths occur annually[7], while *Trypanosoma cruzi* burdens the Americas with 8 million new infections and 10,000 deaths caused by Chagas’ disease every year[8]. To date, the most effective ways to control these parasitic illnesses have been the use of drugs to treat the diseases and insecticides to control the transmission. Due to the spread of resistance to both types of molecules, the search for new agents is a pressing task[9].

This work focused on evaluating the biological activities of different parts of *C*. *nucifera*, from the Punta Patiño Private Natural Reserve, against the parasites that cause Chagas’ disease, leishmaniasis and malaria, and against a laboratory line of breast cancer cells, and to identify the possible molecules responsible for any biological activity.

## Methods

### Ethics statement

Human blood was collected from a pool of volunteers who signed informed consents. This protocol was approved by the bioethical committee of The Gorgas Memorial Institute of Health Sciences in Panama for this study with Number 0001–2017. Eighteen volunteers were recruited and donated sporadically from 2016 to 2018.

*C*. *nucifera* samples were collected from the Patiño Nature Reserve (Chepigana district, Darien region. UTM: 801032 E 913730 N Zone 17P). The authors were provided permission to collect these samples from the owners of the reserve, the National Association for the Conservation of Nature (ANCON), a non-government organization and stakeholder of this study. No other permission was required for research purposes and the field studies did not involve any endangered species.

### Plant material

The husk, leaves, pulp, and milk of the *C*. *nucifera* tree and coconut were collected from their original habitat in the Punta Patiño Natural Reserve in the mostly forested and underdeveloped province of Darien (Panama). The plant was identified by botanist Jose Polanco and voucher samples of the leaves are kept at the Herbarium of the Universidad de Panama; registry number 0111279. The coconut oil was extracted mechanically on location in the Reserve by cutting the coconut meat into small pieces, drying it using gentle oven heating at 60°C and then placing it in a press in a metal tube with slits on the side to force the oil out. The oil was filtered with 0.22 μm membranes (Pall Gelman Acrodisk, Sigma-Aldrich, USA) prior to its use in bioassays. The rest of the parts of the plant was transferred to Panama City for evaluation.

### Preliminary extraction and fractionation

The husk, leaves, kernel, oil and milk were evaluated using small-scale extraction of 10 to 20 g. Samples were macerated in a 2:1 mix of dichloromethane:methanol overnight; these extracts were vacuum filtered and collected in round bottom flasks. This step was repeated several times until an exhaustive extraction was accomplished (approximately 5 to 7 times each). To obtain fractions, samples were passed through a C-18 SepPak column (Waters Corporation. Milford, MA, USA) with a methanol water gradient, starting at 50:50 ratios and ending in 100% methanol. Seven fractions were collected at 50:50, 60:40, 70:30, 80:20, 90:10, 100% methanol and 100% dichloromethane and dried by rotary evaporation. All samples were dissolved in Dimethyl sulfoxide (DMSO) (Sigma-Aldrich, USA) for bioassays.

### Large scale extraction

Two aliquots of 15 g each of fresh leaf pieces (approximately two leaf blade’s worth of material) were brought to a boil in 250 ml of distilled water, and left boiling for 5 or 15 min. Both samples were brought to room temperature and filtered, first through 0.8 and then through 0.22 μm membranes before testing. The concentration of the aqueous extract was calculated by drying 10 ml and measuring the weight, which corresponds to a final concentration of ~70 μg/ml in 10% v/v of the decoction. 30 ml of sample were kept for further tests while the remaining amount was lyophilized. Lyophilized samples were resuspended in freshly boiled demineralized water to allow for originally active molecules in the infusion to be dissolved in the same conditions in which they were extracted, after which they were filtered through 0.22 μm membranes before testing.

Additionally, 450 g of fresh leaf pieces were macerated in a 9:1 mix of methanol: water and these extracts were vacuum filtered and collected in round bottom flasks. This step was repeated several times until an exhaustive extraction was accomplished (approximately 5 to 7 times each).

### Solid phase fractionation

The aqueous extract of the leaves and the methanol water extract were subjected to solid phase extraction to yield several fractions for further testing. A modification of the procedure described by Bianchi et al (2015)[10], using solid phase extraction (SPE) cartridges, was performed. The C-18 SPE cartridges (SUPELCO, Sigma-Aldrich, USA) were preconditioned first with methanol and later with distilled water. Back to back elutions with distilled water, ethyl acetate and methanol were performed, in this order, to produce fractions F0, F1, and F2 for each extract (a flow through fraction was included for the aqueous and methanol water extracts). All fractions were dried in a rotary evaporator until further use.

### Tandem mass spectrometry fractionation

*C*. *nucifera* extracts were analyzed following the methodology by Chang et al (2011)[11] with modifications using a Bruker Daltonics micrOTOF-QIII mass spectrometer (Bruker, Bremen, Germany) with atmospheric pressure chemical ionization (APCI) source in positive mode. The HPLC consisted of an Agilent Technologies (Waldbronn, Germany) 1290 Infinity with a binary pump, an autosampler, a diode-array detector on the 190–400 nm absorption region and a Kinetex Phenyl-Hexyl (2.1 x 50 mm, 1.7 μm, Phenomenex, Torrance, USA) column. The binary phase consisted of 0.1% formic acid in water (grade milli-q) (A) and methanol (grade LC-MS) (B) at the flow rate of 0.3 ml min-1. The elution profile was: 0–6 min, 20–65% B in A (linear gradient); 6–9 min, 65–100% B in A (linear gradient); 9–15 min, 100% B in A (isocratic; column wash); 15–18 min, 100–20% B in A (linear gradient; return to initial conditions). Positive ion mode APCI conditions were: corona current 5000 nA, capillary voltage 4000 V with the end plate offset at 500 V, drying gas (N_2_) temperature 300°C with the flow rate of 3.0 L min-1, nebulizer gas (N_2_) pressure 2.0 Bar, vaporizer temperature if 300°C and mass range for data acquisition 50–3,000 Da.

### Molecular networking

The raw MS/MS data was analyzed by molecular networking using the Global Natural Products Social Networking (GNPS) platform. In summary, raw data was converted to mzXML file for mass spectral molecular networking, parameters for cosine scores, precursor mass tolerance (Da), fragment mass tolerance (Da), matched peaks, consensus spectra for cosine score and parent mass tolerance (Da) were set using the global natural products social molecular networking platform (GNPS, https://gnps.ucsd.edu). Each MS/MS spectra were clustered with MS-Cluster with a parent mass tolerance of 1 Da and a MS/MS fragment ion tolerance of 0.3 Da. The spectra in the network were then searched against GNPS's spectral libraries resulting in the dereplication of several molecular families of compounds. Raw data results are available at GNPS through the accession ID = 9bd8313eec10453bb85a13134205e98c. The resulting similarity matrix was analyzed with Cytoscape 3.5.1 where each consensus spectra was visualized as nodes and their similarity cosine as edges. The color of the nodes informs the source of the precursor ions, and the edge thickness corresponds to the cosine similarity score, where thicker lines correspond to higher similarity[12].

### Bioassays

At least two independent experiments were performed in duplicate in 96-well plates with each sample having a final concentration of 10 μg/ml per well. In the case of the oil sample, 10 μg/ml w/v was used. As a negative control, DMSO was utilized to calculate 100% growth, and wells with only media and extracts, fractions, oil or aqueous extracts were used to eliminate any possible intrinsic fluorescence of the samples. To test the activity of the aqueous extracts, 2.5% to 10% of the total sample volume in the wells was replaced with the infusions. The same percentages of demineralized water were used as a negative control. All parasites were obtained from the Walter Reed Army Institute of Research, MD, USA). Cell lines Vero and MCF7 were obtained from the American Tissue and Cell Collection (ATCC No. CRL-1587 and ATCC HTB22, respectively).

### Anti-trypanosoma assay

*T*. *cruzi* bioassays were performed using a colorimetric method; the inhibition of parasite growth was assessed by the expression of the reporter gene for beta-galactosidase (β-Gal) in the recombinant Tulahuen clone C4 of *T*. *cruzi*[13]. Assays were performed on the intracellular amastigote form of the parasite infecting African green monkey kidney (Vero) cells, which were exposed to the test samples during 120 h at 37°C under an atmosphere of 5% CO_2_/ 95% air. The resulting color from the cleavage of chlorophenol red-β-D-galactoside (CPRG) (Sigma-Aldrich, USA) by β-Gal expressed by the parasite was measured at 570 nm. Benznidazole, at 10, 1 and 0.1 μg/ml producing an IC_50_ around 3.8 μM, was used as the positive control drug following the guidelines of the Drugs for Neglected Diseases initiative (DNDi) for the study of *T*. *cruzi*[14].

### Anti-leishmania assays

The anti-leishmania activity was evaluated following the protocol described by Calderon et al, 2006[15], using the fluorescent DNA intercalator PicoGreen (Invitrogen, USA). The species responsible for visceral leishmaniasis, *L*. *donovani*, was used for the assays. For each biosassay, 1×10^6^ cells were placed in each well of a 96-well plate with the extracts in a final volume of 100 μl and incubated for 3 days. Amphotericin B was used as the positive control where the typical IC_50_ response of *L*. *donovani* to this drug is 70–120 ng/μl. A PicoGreen cocktail was added at a 1∶4 dilution and incubated at room temperature for 5 min before fluorescence was measured at 485 nm.

### Anti-cancer assays

Cytotoxic activity against MCF-7 (human breast cancer cell line) was performed following the standard protocol of the National Cancer Institute, using the metabolic reduction of 3-(4,5-dimetylthiazol-2-yl)-2,5-diphenyltetrazolium bromide (MTT) (Sigma Aldrich, USA) by the mitochondrial succinate-dehydrogenase as a measure of viability of the cells[16]. MCF-7 cells were seeded (4 × 10^4^ cells per well) in a final volume of 100 μl/well in 96-well plates and incubated with Roswell Park Memorial Institute medium (RPMI-1640,Sigma-Aldrich, USA) supplemented with gentamicin (0.05 mg/ml), L-glutamine (Gibco, Invitrogen, Carlsbad, CA, USA; 2 mM), NaHCO3 (Sigma-Aldrich, USA 4.6 mM), HEPES buffer (Sigma-Aldrich, USA 25 mM), and FBS (10%) (Gibco), at 37°C. After attachment, they were treated with test samples. After incubation at 37°C for 72 h, the cells were fixed with cold trichloroacetic acid at 20% (w/v) for 2 h and stained with Sulforhodamine B (SRB) (Sigma-Aldrich, USA) dye for 30 minutes. The protein-bound dye is dissolved in Tris base solution for OD determination at 540 nm on an ELISA Plate Reader. Adriamycin diluted in DMSO was used as the positive control (normal IC_50_ value 20–50 nM).

### Anti-plasmodium assays

Activity against the causative agent of malaria was performed by culturing human erythrocytes and infecting them with *P*. *falciparum*, as described by Trager and Jensen, 1976[17]. Briefly, the W2 (Chloroquine resistant) and 3D7 (Chloroquine sensitive) strains of *P*. *falciparum* were cultured in RPMI 1640 medium (Sigma-Aldrich, USA) supplemented with 10% human serum (from O+ blood) at a hematocrit of 2% erythrocytes (O+) at 37°C in a gas mixture of 5% CO_2_, 5% O_2_, and 90% N_2_. Human blood was collected from a pool of volunteers which signed informed consents. This protocol was approved by the bioethical committee of the Gorgas Memorial Institute for the Health Sciences for this study with Number 0001–2017. Eighteen volunteers were recruited and donated sporadically from 2016 to 2018. Parasites were synchronized by a temperature cycling technique to enrich the culture with parasites in the schizont stage to use for the assays as described by Almanza et al, 2010[18]. 180 μl of parasite culture and 20 μl of the samples were transferred to 96-well plates and incubated for 24 h. After this period, a mix of the PicoGreen DNA fluorescent dye (Invitrogen, USA) was added to a final concentration of 1% and after 30 min incubation the signal was read on a fluorescence plate reader as described in Corbett et al, 2004[19]. Alternatively, in some assays, after a 24 h incubation period, parasitemia was recorded using Hoechst 33342 fluorescent dye (Invitrogen, USA) to a final concentration of 2 μg/ml. Chloroquine was used as a positive control. The concentration of the aqueous extract was calculated by lyophilizing 10 ml and weighing it, which gave a final concentration of 70 μg/ml in 10% v/v of the decoction.

### Cell viability assay

The assays were based on those described by Mosmann et al [20]. Briefly, Vero epithelial cells were seeded (5x10^3^) in a final volume of 100 μl/well on 96-well plates using RPMI-1640 medium (Sigma-Aldrich, USA), L-glutamine (Gibco, Invitrogen, Carlsbad, CA, USA; 2 mM), NaHCO3 (Sigma-Aldrich, USA, 4.6 mM), HEPES buffer (Sigma-Aldrich, 25 mM)supplemented with 10% fetal bovine serum (Gibco, Invitrogen, USA) and 1% penicillin/streptomycin. The cells were allowed to grow for 24 hours before adding the samples of aqueous extracts at the same concentrations as the antiplasmodial assay in volume percentages (v/v). A negative control, without any sample, was placed in all plates and counted as 100% growth. Adriamycine was used as the positive control. All samples were incubated for three days before staining and examining for the reduction of 3-(4,5-dimethylthiazol-2-yl)-2,5-diphenyltetrazolium bromide (MTT) after incubating it with the cells for a period of four hours. The ELISA plate reader was used at a wavelength of 570 nm.

### Quantification of parasitemia using flow ytometry

Parasitemia was obtained by incubating parasitized red blood cells with Hoechst 33342 (Invitrogen) at 2 μg/ml for 30 minutes while protected from light. After staining, samples were fixed using formaldehyde at 1%. Acquisition was performed on a PARTEC CyFlow Space cytometer equipped with a UV laser. The background staining of an uninfected red blood sample was always subtracted[21].

### Microscopy

For microscopic verification of invasion, thin smears for all samples were made and stained with Giemsa (Sigma). Parasites in 1,000 red blood cells were counted with the microscopist blinded to the identity of the samples[21].

### Statistics

The data was analyzed with the MS Office Excel AddOn, LSW Toolbox and the GraphPad Prism 6 softwares performing one-way ANOVA, Bonferroni's Multiple Comparison Test and student t test. The significance level was set at 5%.

## Results

The chemical organic extraction process of a 2:1 mix of dichloromethane:methanol from the husk, meat, leaves and milk of *C*. *nucifera* yielded an abundant amount of material for each sample as shown in (S1 Table). Organic extracts were evaluated against the tropical parasites *T*. *cruzi*, *L*. *donovani*, and *P*. *falciparum* and against the breast cancer cell line MCF-7, all at 10 μg/ml. The trypanosomatids and the cancer cells did not respond to the presence of the extracts or the oil, when compared to controls (Table 1); however, a moderate activity was detected against the apicomplexan responsible for human malaria. The growth for *Plasmodium* in this initial screening was recorded as 46.1% for the leaf extract and 82.9% for the oil, while the husk, milk and kernel failed to alter the normal development of the parasite by more than 17%. After obtaining these results, a further organic fractionation of the leaf extract was performed with the aim of isolating the molecules responsible for the anti-Plasmodium activity. Seven fractions from the additional purification of the leaf extract were tested against *P*. *falciparum* at a concentration of 10 μg/ml, failing to show activity above 6% parasitemia inhibition (S2 Table).

**Table 1:** Anti-trypanosomatid and anticancer activity of organic extracts of different components of *Cocos nucifera* from Punta Patiño.

	% Growth*			
Source	*L*. *donovani*	*T*. *cruzi*	MCF-7	*P*. *falciparum*
Leaf	100	94.3	100	46.1
Husk	98	93.6	100	96.5
Meat	100	95.8	100	93.2
Milk	97.9	100	100	88.3
Oil	100	100	100	82.9

*% Growth is calculated with respect to controls with DMSO which are considered at 100% growth. The results are the means of two experiments with two replicates each.

**Fig 1:**
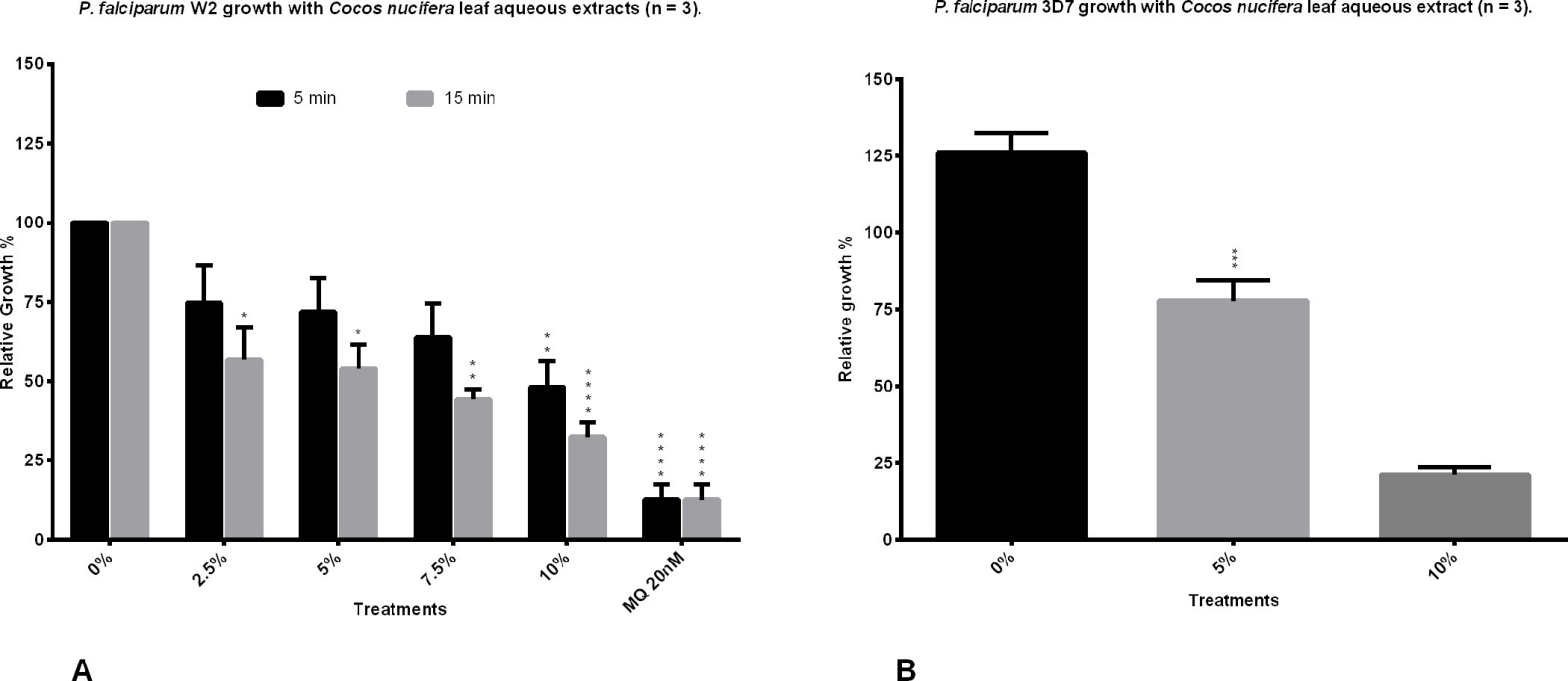
Anti-Plasmodium activity of the aqueous leaf extracts of *Cocos nucifera*. A) Coconut palm leaves were boiled for 5 or 15 min in water and added to *P*. *falciparum* W2 cultures by triplicates at different volume percentages. B) The 15 min water decoction was added to *P*. *falciparum* 3D7 cultures at different volume percentages. The growth of the parasites was analyzed by flow cytometry and compared to culture controls with only water added which were set as the 0% of extract volume. Mefloquine at 20 nM was used as positive control. Results shown are the mean of three experiments ±SEM values. Significance values were calculated at alpha 0.05. * = p<0.05, ** = p<0.01, *** = p<0.005.

To analyze an extract which would more closely resemble the native usage of the plant, leaves were subjected to a simple boiling treatment in water and two different boiling times were evaluated. When 15 g of leaves were boiled for 5 and 15 min, and the aqueous extracts added at a 10% v/v, there was activity against the chloroquine resistant W2 strain of *P*. *falciparum* parasites, causing the growth to be 48% and 32% those of the controls, respectively (Fig 1A). The extract which was boiled for 15 minutes was chosen for further investigation beginning with a test against the chloroquine sensitive 3D7 strain of the malaria parasite. It showed a strong activity against the latter, causing the relative growth to be 26% that of the controls at the maximum concentration used (Fig 1B).

**Fig 2:**
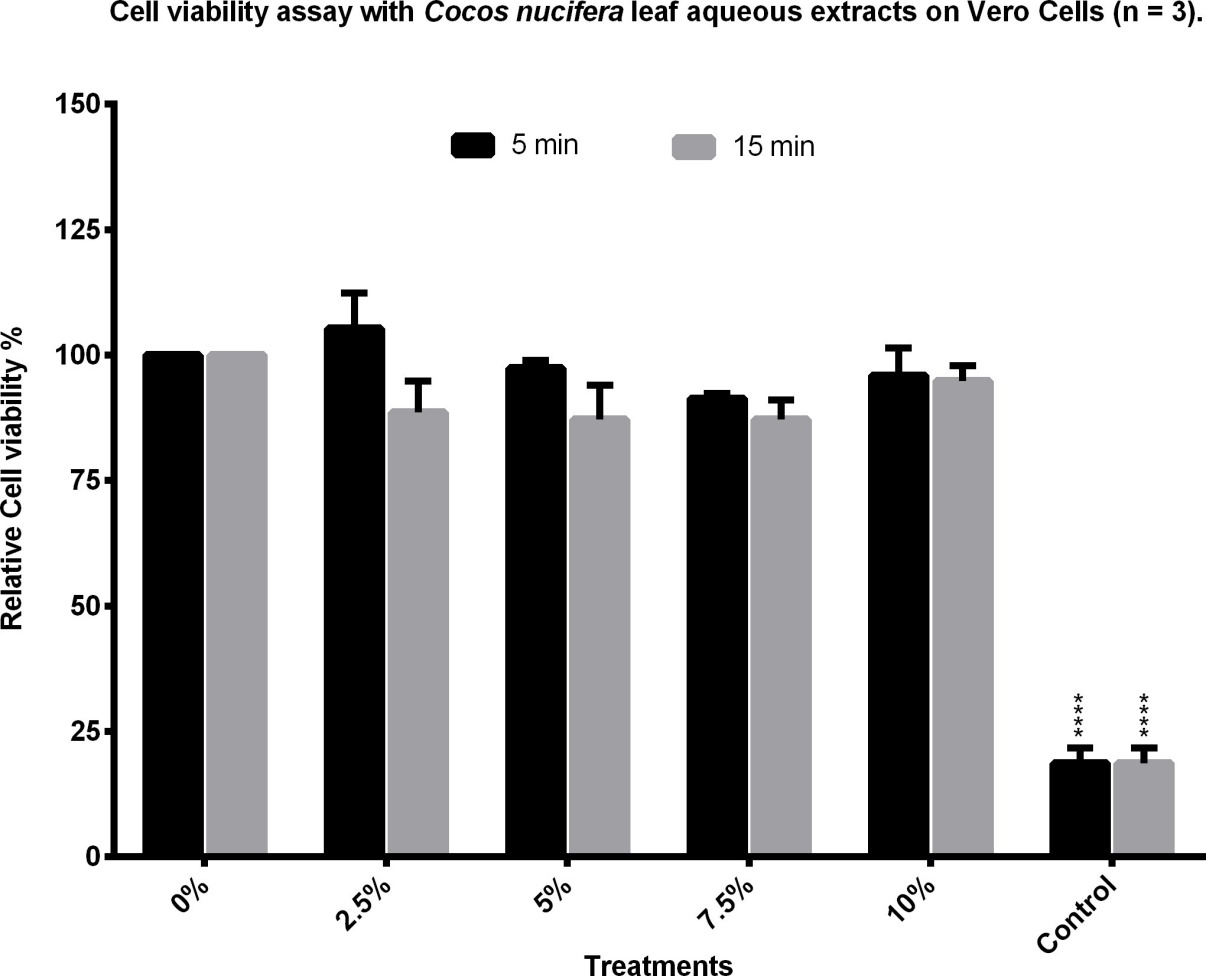
Cytotoxic effects of the aqueous leaf extract of *C*. *nucifera*. Coconut palm leaves were boiled for 5 or 15 min in water and added to Vero cell cultures at different volume percentages. The toxic effect of the extracts was analyzed by MTT assays and compared to controls with only water added. Adriamycine at 4 μg/ml was used as positive control. Results shown are the mean of three experiments ±SEM values.

**Fig 3:**
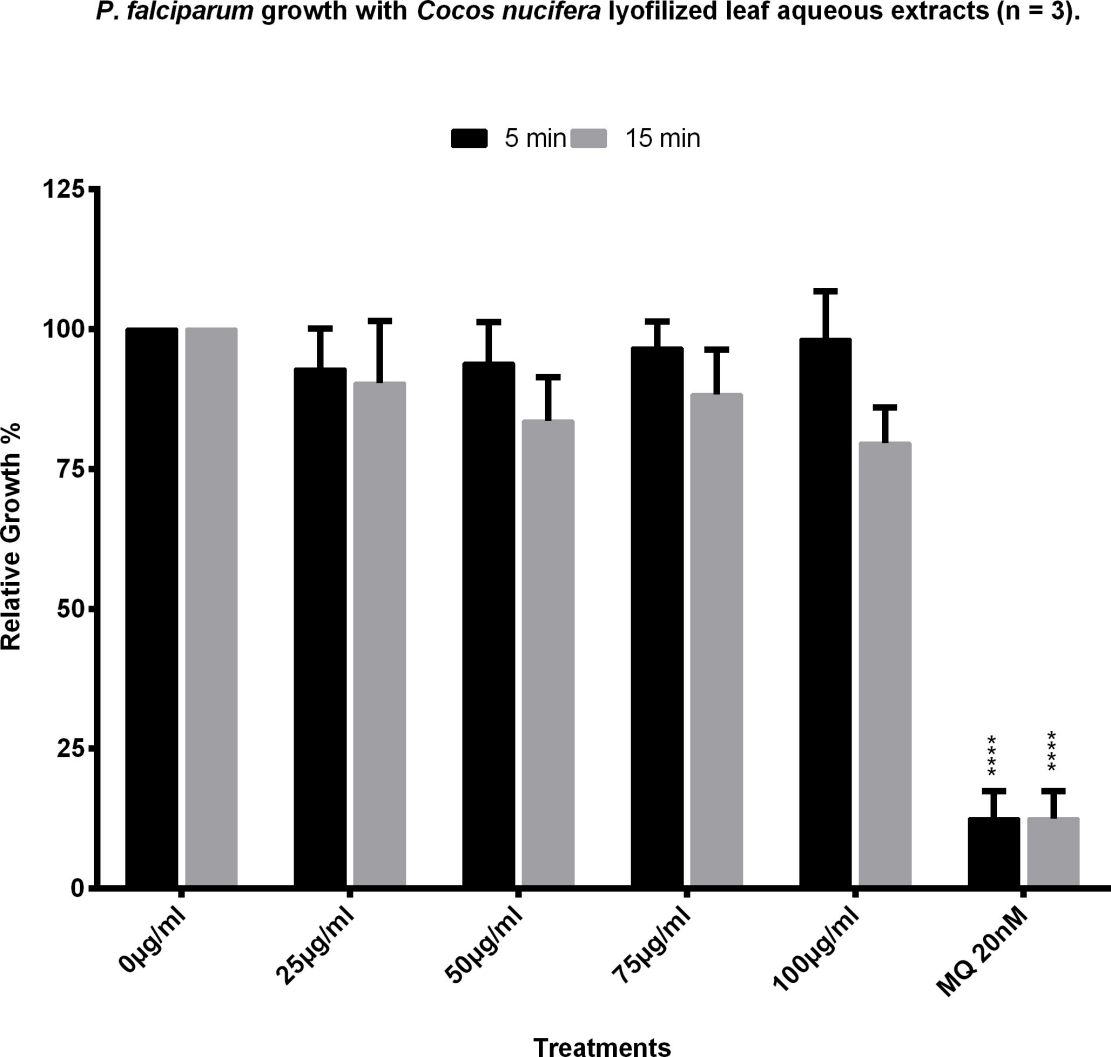
Anti-Plasmodium activity of the lyophilized aqueous leaf extracts of *Cocos nucifera*. Aqueous extracts of coconut palm leaves were lyophilized, resuspended in water and added to *P*. *falciparum* cultures at different concentrations. The growth of the parasites was analyzed by flow cytometry. Control cultures with only water added were considered as having 100% growth. Mefloquine at 20 nM was used as positive control. Results shown are the mean of three experiments ±SEM values.

The aqueous extracts were tested for toxicity against Vero cells and, when compared to a control with only water, (set as 100% cell viability) all samples showed a cell growth of over 80% (Fig 2). This extract was subjected to lyophilization and resuspension in boiling water to make sure they dissolve in the conditions in which they were extracted. After resuspending and filtering, the lyophilized product was tested on the parasites at 100, 75, 50, and 25 μg/ml. None of these concentrations had any significant effect on the parasites when compared to the controls (Fig 3). Lyophilized samples were also tested for toxicity on Vero cells and they showed no cytotoxic effects.

To understand why the lyophilized product had lost its activity, other technologies were used for drying the aqueous (nitrogen gas flow, rotary evaporator and speed vacuum) to see if the activity was preserved. In all instances, confronted with *P*. *falciparum*, the activity was lost (data not shown). To examine if the composition of the extract had changed during the process, an HPLC analysis of both fresh and dry products were separately run. The composition profile of the fresh decoction changed after being subjected to lyophilization S1 Fig.

**Fig 4:**
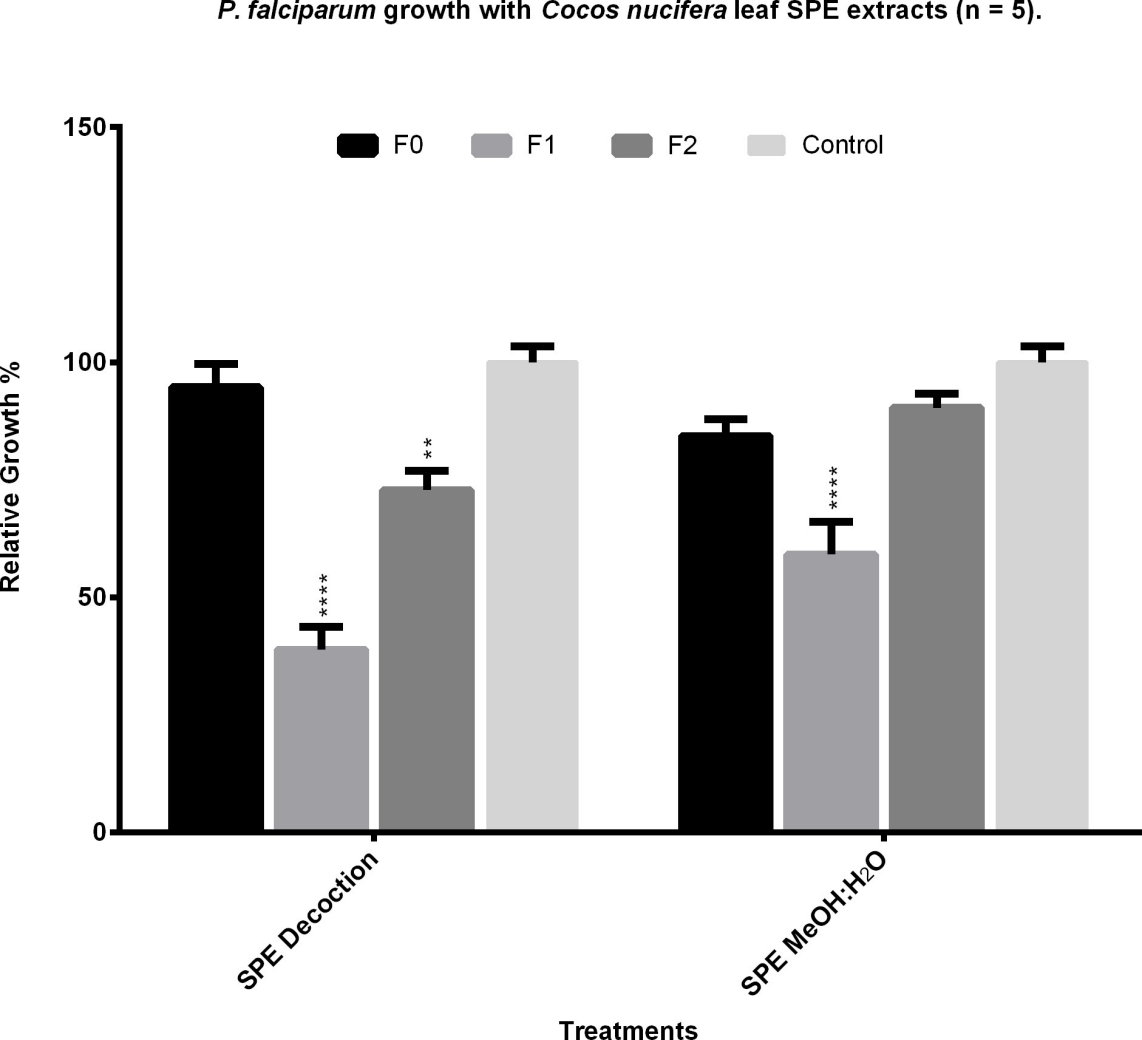
Antiplasmodial activity of the SPE fractions of the aqueous and organic extracts of the leaves of *C*. *nucifera*. Solid phase extraction was performed on crude extracts from leaves subjected to a decoction for 15 minutes (SPE Decoction) and on extracts from a 9:1 mix of methanol and demineralized water extraction (SPE MeOH:H_2_O). Back to back elutions with distilled water, ethyl acetate and methanol were performed, in this order, to produce fractions F0, F1, and F2. Fractions were added to *P*. *falciparum* W2 cultures by triplicates at 100 μg/ml. The growth activity of the extracts was assessed by microscopy and compared to culture controls with only water added. Results shown are the mean of five experiments ±SEM values. **** = p<0.005 as compared with controls.

**Fig 5:**
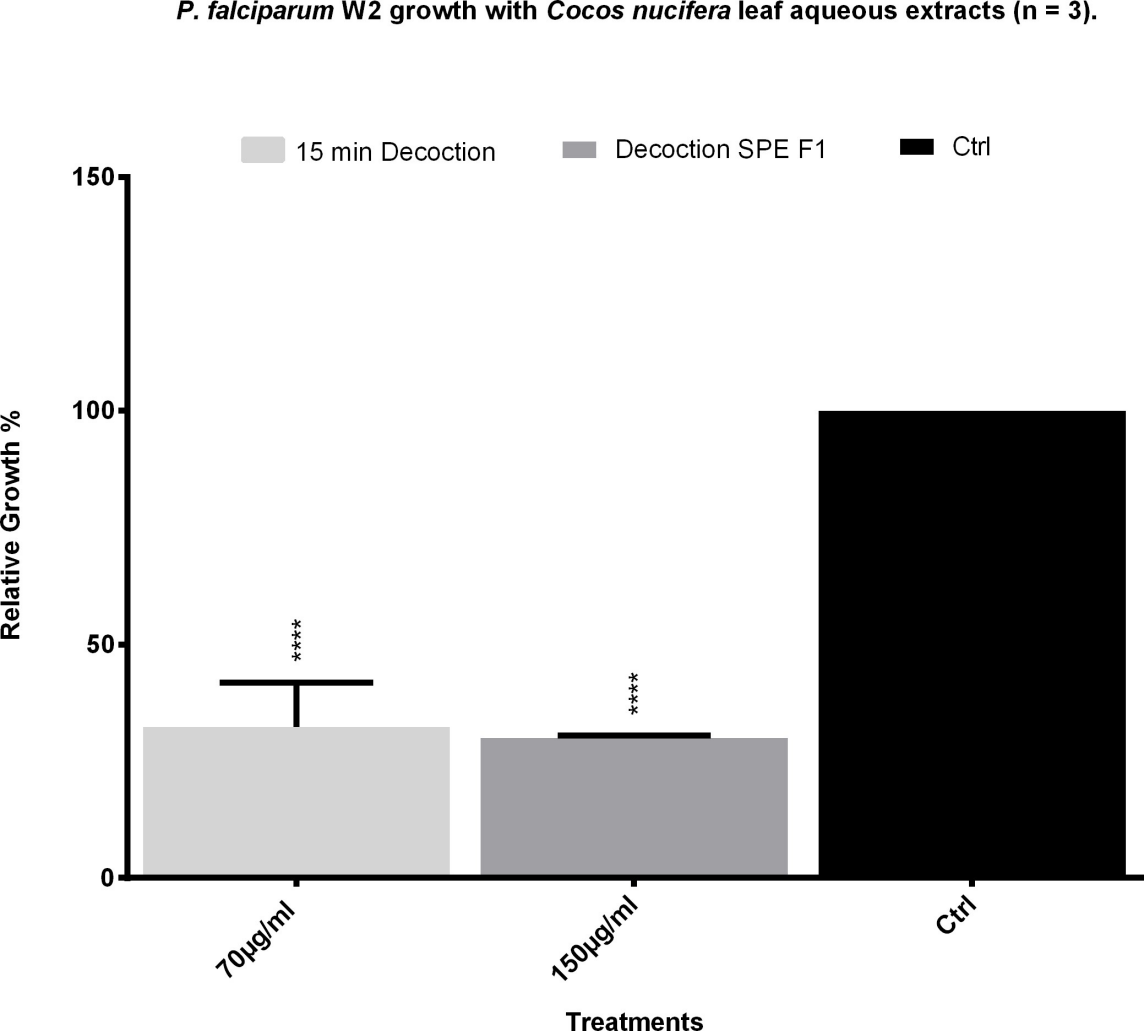
Comparison of the efficacy of a fraction vs the whole decoction of the leaves of *C*. *nucifera* against *P*. *falciparum*. F1 of the SPE fractionation was compared with the decoction at double the concentration of its mother mixture. The graph shows the result of three experiments run in duplicates.

To test if another method of extraction would yield more activity than the aqueous samples, a methanol:water (9:1) mix was used to obtain another extract. Both the aqueous and the methanol:water extracts were fractionated separately using SPE cartridges. The activity of the fractions obtained was tested, with F0 and F2 from both extraction procedures showing a very modest action against the parasites, while F1 seemed to retain the majority of the activity showed in the fresh 15 min decoction. The organic extract also showed similar activity in all three SPE fractions, although less than the aqueous one (Fig 4). The activity of Fraction 1 of the SPE was compared to that of the decoction, noting that part of the activity was lost since less than half of the concentration of the decoction showed about the same inhibition of parasitemia than the fraction (Fig 5).

**Fig 6:**
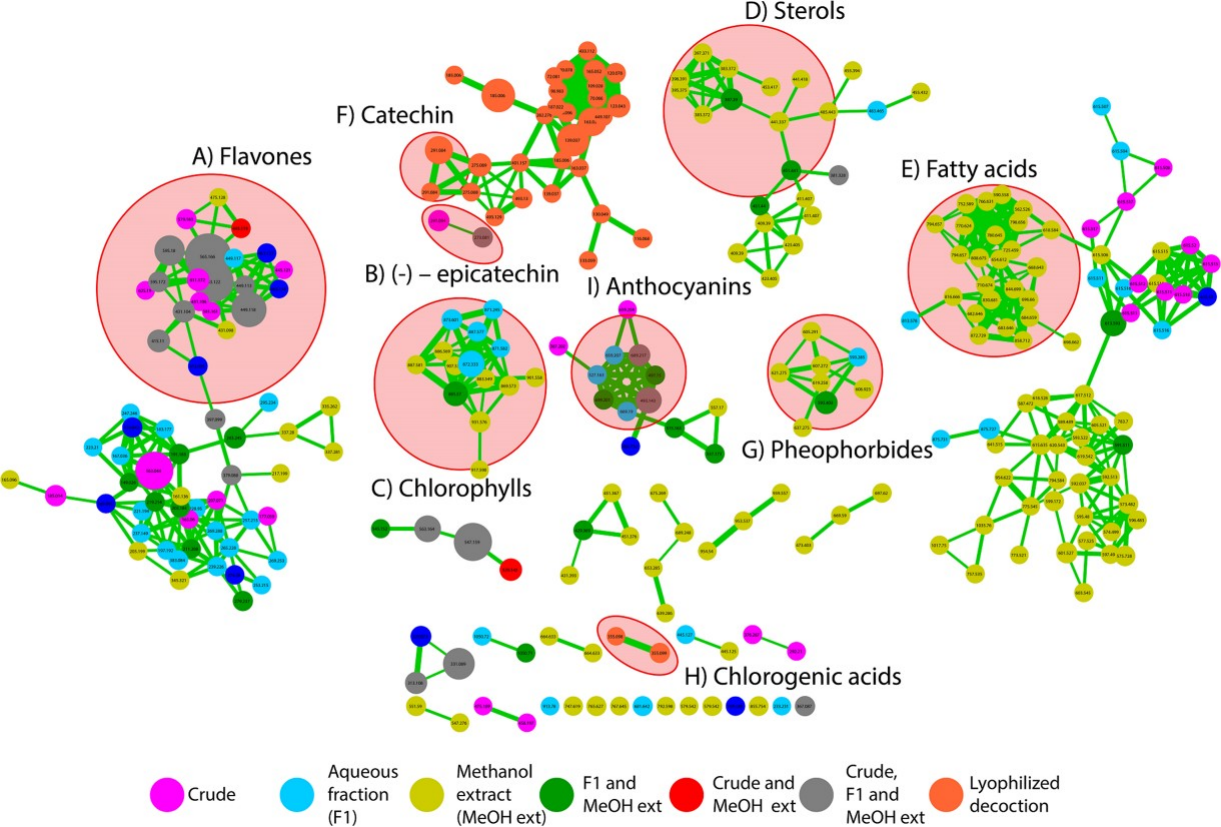
Molecular network of leaves of *C*. *nucifera* aqueous extracts and active fractions. A molecular network of coconut leaves water extracts and SPE fractions was created using the online workflow at GNPS. A) Flavones clusters containing nodes of compounds of interest. B) (-)–epicatechin cluster. C) Chlorophyll cluster. D) Sterols cluster. E) Fatty acids cluster. F) Catechin cluster. G) Pheophorbide A cluster. H) Chlorogenic acid cluster. The color of the nodes informs the source of the precursor ions, and the edge thickness corresponds to the cosine similarity score, where thicker lines correspond to higher similarity. The CYMK colors in magenta correspond to compounds present in the crude aqueous leaf extract, nodes in cyan correspond to compounds present in the active fraction of the crude extract and nodes in yellow correspond to compounds present in the methanol extract. Nodes in orange correspond to compound hits from the lyophilized decoction.

To study the detailed chemical identity of the compounds present in the active samples they were analyzed with HPLC ESI-Q-TOF mass spectrometry. The results were dereplicated with MS/MS-based molecular networking using the online workflow at GNPS (http://gnps.ucsd.edu/ProteoSAFe/status.jsp?task=9bd8313eec10453bb85a13134205e98c). The resulting network contains two hundred forty-five (245) nodes, clustered in nineteen molecular families and 14 individual nodes, after filtering nodes from blanks (Fig 6). We dereplicate several molecular families such as sterols, fatty acids, chlorophyll, and flavanols. The dereplication of the MS/MS data resulted in fourteen compounds from decoction, three from lyophilized decoction, six from decoction F1, eight from MeOH F1 clustered with several unidentified compounds as well (Table 2, Fig 6).

**Table 2:** LC-MS/MS analysis of the 15 min decoction of *C*. *nucifera* leaves. The list shows only those compounds that were dereplicated.

Origin	GNPS Libraries Compounds Match	Consensus Spectra (Da)	Library (Da)	Adduct	Cosine	Mass Diff	Shared Peaks
**Decoction**	**Malvidin 3-O-galactoside**	493.1430	493.1330	Cation	0.97119	0.0100098	8
	**(-)-Epicatechin**	291.0940	291.0870	[M+H]+	0.956362	0.00698853	6
	**Hexanoside of (iso)vitexin**	595.1800	595.1730	M+H	0.951231	0.00701904	12
	**Hesperidin methyl chalcone**	625.1900	625.2130	[M+H]+	0.635788	0.0230103	7
	**Paniculatin**	595.1760	595.1650	M+H	0.645329	0.0110474	12
	***C*-Hexosyl-chrysoeriol**	463.1330	463.0000	[M+H]	0.721265	0.132996	8
	**Peonidin 3-galactoside**	463.1320	463.1230	Cat	0.91611	0.00900269	4
	**Vitexin**	433.1220	433.1130	M+H	0.915217	0.00900269	11
	**Sterol derivative**	387.2020	387.3630	[M+H]+	0.78407	0.161011	5
	**Isoorientin**	449.1180	449.1080	M+H	0.871378	0.0100098	10
	**O,C-pentosyl-hexosyl-apigenin**	565.1660	565.0000	[M+H]	0.662414	0.166016	6
	**Isovitexin**	415.1100	415.1020	M+H-H2O	0.733261	0.00799561	7
	**C-Hexosyl-luteolin O-hexoside**	611.1720	611.0000	[M+H]	0.831562	0.171997	8
	**2''-O-beta-D-Xylopyranosylorientin**	581.1610	581.1500	M+H	0.820408	0.0109863	11
**Lyophilized Decoction**	**Chlorogenic Acid**	355.0990	355.1030	[M+H]+	0.85533	0.0039978	5
	**Catechin**	291.0840	291.0820	M+H	0.71072	0.00201416	26
	**trans-5-O-Caffeoylquinic acid**	355.0980	355.0950	M+H	0.854506	0.00299072	4
**F1 from Decoction**	**2-Propenoic acid**	557.1730	556.2020	M+NH4	0.604252	0.970947	5
	**beta-Sitosterol**	397.3710	397.6000	[M-H2O+H]+	0.76585	0.229004	13
	**Pheophytin**	871.2450	871.5900	M+H	0.940284	0.345032	11
	**Isoorientin**	449.1170	449.1080	M+H	0.816247	0.00900269	8
	**Triacylglycerol (18:1/18:3/18:3)**	875.7270	876.7210	M+NH4	0.656969	0.994019	16
	**Pheophorbide A**	593.4030	593.2690	M+H	0.930512	0.134033	11
**F1 from MeOH**	**Monogalactosyldiacylglycerol (18:2/18:3)**	775.5450	776.5440	M+NH4	0.643473	0.999023	7
	**Diacylglycerol (16:0/18:1/0:0)**	593.5220	594.5223	M+NH4	0.639393	1	9
	**Stigmasterol**	395.3750	395.5000	[M-H2O+H]+	0.638058	0.125	12
	**Campesterol**	383.3720	383.4000	[M-H2O+H]+	0.718911	0.0279846	11
	**2-Linoleoyl-1-palmitoyl-sn-glycero-3-phosphoethanolamine**	716.5300	716.5200	M+H	0.648956	0.0100098	7
	**Sphingolipid GalCer(d18:2/20:1)**	752.5890	751.5960	M+H	0.938653	0.992981	23
	**(+)-.alpha.-Tocopherol**	431.3930	431.3780	M+H	0.91453	0.0150146	4
	**Chlorophyll b from Chlorella**	907.5330	906.9500	[M+H]	0.787933	0.583008	6

*The 15 min decoction of *C*. *nucifera* leaves was analyzed by LC/MS–MS TOF spectrometry. The list shows only those compounds that were identified using the GNPS platform

## Discussion

The products of *C*. *nucifera* have been attributed with a diverse array of healing properties, from anti-inflammatory to anti-hypertensive, analgesic, and antibacterial activity[22]. We tested husk, pulp, leaves and milk of *C*. *nucifera* on parasites and a breast cancer cell line resulting in a moderate activity of the leaf against the *P*. *falciparum* parasite. The low activity of the husk in our hands contrasts with findings in the literature. This discrepancy might be due to different specific environmental conditions of both coconut plantations which could elicit the production of different metabolites. As for the leaves of *C*. *nucifera* from Punta Patiño, they showed a moderate activity against *P*. *falciparum* that deserved further investigation.

The organic fractionation of the leaf extract resulted in a loss of the activity. For this reason, a water extraction was tested, revealing that 15 min of boiling yields an interesting activity against the asexual stage of the malaria parasite.

Non-toxic effects of the aqueous extract on Vero cells were obtained at volume percentages equal to 70 μg/ml, the same concentration at which the extract inhibited parasitic growth: the decoction did not affect the viability of the epithelial cell line by more than 20%, at the highest volume percentage used. This observation opens the possibility, pending further research, that the ingestion of an infusion from the leaves would not cause severe adverse effects in living organisms.

The loss of the antiparasitic activity upon lyophilization was surprising, but the comparison of the HPLC chromatograms of our liquid extract vs its lyophilized form show a difference in their composition. Additionally, when we carried out the molecular networking analysis based on the comparison of MS/MS data, to cluster compounds in molecular families according to their spectral similarities using the GNPS platform, the results revealed the presence of different compounds in each sample; for example, epicatechin and its derivatives in the fresh decoction while catechin, caffeoylquinic acid and chlorogenic acid are detected only in the lyophilized decoction (Table 2). It seems that the components of the extract could suffer structural modifications or degradation when they are submitted to drying processes. A noteworthy example of this change is the epimerization of tea catechins which is thought to contribute to the turnover from nonepi- to epicatechin in some tea samples that have been tested at different temperatures[23]. The leaf samples may contain catechin which is epimerized into epicatechin when they are boiled. However, when the temperature is drastically lowered as is the case in lyophilization (-80°C), the epimerization could be reverted to catechin. The MS/MS technique does not allow for the differentiation between epimers, however, the presence of (-)-Epicatechin in the decoction and its absence from the lyophilized decoction cannot be ignored. Furthermore, the HPLC profile data unequivocally shows an evident difference in the composition of both samples which supports this idea (S1 Fig). Another explanation for the inactivity of the sample after drying could be found in the loss of volatile compounds needed to affect the parasite. Arguably, the F1 fraction of the SPE retains a good amount of the activity, pointing out to the possible influence of other components of the extract, retained in F0 and/or F2, influencing in some type of degradation or change exerted in one or more of the active components. Whichever the explanation, the chromatograms with different HPLC profiles (S1 Fig), the different MS data (Table 2) and the difference in activity of the liquid vs the lyophilized extract (Fig 3) leave little doubt that there is a change in the composition of the decoction or in the chemistry of the components of the extract after drying.

In general terms, the molecular families we have found in the molecular networking analysis are flavones (isoorientin, apigenin, vitexin, isovitexin, and luteolin), sterols, fatty acids and chlorophyll (Table 2). Of the flavones, apigenin, vitexin, isovitexin, and luteolin have been found to be used against malaria[24,25]

In the sterols, stigmasterol and β–stigmasterol have been found in other studies of antiplasmodial plants, however their bioactivities are poor when tested by themselves, suggesting in all cases the possibility of a synergistic effect with other component(s) of the extract[26].

Chlorophyll subproducts pheophorbide a, pheophorbide derivatives, and pheophytin have also been described in other studies as having several biological activities. More recently, in a report by Jansen et al (2017) it is described the moderate activity against *Plasmodium* parasites[27] of a pheophorbide a–related compound identified as 13b-hydroxypheophorbide a, which plays an important role in photosynthesis. These compounds (pheophorbide and pheophytin) are found notably absent from the active decoction, the inactive lyophilized decoction, and the F1 fraction from the MeOH fractionation but, present in the active F1 fraction from the active decoction, which makes them candidates for further studies. It is possible that they were undetected in the active decoction because of their strong affinity for the eluent solvent used for F1.

The merozoite surface protein 2 (MSP2) from *P*. *falciparum* is abundant on the surface of the merozoites. It is not structured and forms amyloid-like fibrils in solution. Chandrashekaran et al (2011) found that epigallocatechin gallate (EGCG) can alter the β-sheet-like structure of the fibril and disaggregate pre-formed fibrils of MSP2 into soluble oligomers[28]. Our antimalarial assays are performed on the mature stage of the parasite when the release of merozoites takes place in the presence of our extract. It is quite possible that MSP2 is affected by a flavonoid compound or several of the flavonoid compounds present in our active samples, causing an inability of the merozoites to invade uninfected erythrocytes.

Literature shows that the flavone luteolin-6-C-glucoside is a molecule that is an inhibitor of the *P*. *falciparum* M18 Aspartyl Aminopeptidase (PFM18AAP). This aminopeptidase is found in all intra-erythrocytic stages of the parasite, and functions to complete the hydrolysis of host hemoglobin into amino acids for use in de novo protein synthesis by the parasite[29]. Having this molecule present in the most active samples of the decoction and the F1 from the decoction while missing or in undetectable levels in the less active F1 methanol extraction and the completely inactive lyophilized extract, it is possible that this is the molecule which is largely responsible for the antiplasmodial activity observed in our study. However, further experiments are required to validate this hypothesis.

The MS data show some compounds that could be found in the active samples and which could be analyzed in the future to try to isolate the antiplasmodial activity, but a synergy between two or more compounds of the extract is the most plausible scenario of our antiplasmodial activity.

In summary, the compounds we found (flavones, isoorientin, apigenin, vitexin, isovitexin, and luteolin) in the leaves of *C*. *nucifera* from Punta Patiño, Panama, are the same as the ones tested by several research groups, originating from other natural sources, against the malaria causing parasite *P*. *falciparum*. This is the first report describing the antimalarial activity of leaves from *C*. *nucifera* on *P*. *falciparum* parasites and the use of advanced dereplication techniques to identify the possible secondary metabolites responsible for it. Their activities have been described and mechanisms of action related to fatty acid synthesis, hexose transporters, adhesion molecules and peptidases are suggested.

## Conclusions

Our study evaluated the antiparasitic and anticancer activity of some components of the coconut plant. We have shown that 10 μg/ml of a 2:1 dichloromethane:methanol leaf extract is capable of specifically inhibiting the growth of the parasite responsible for the most deadly form of human malaria, *P*. *falciparum*, to at least half that of the controls. Nonetheless, when this organic extract was fractionated to further isolate the activity, the effect on the parasite was lost, suggesting a possible synergistic action by two or more components of the original extract. The aqueous extract of the leaves, which could be easily prepared by the local inhabitants in remote places like the Punta Patiño Natural Reserve in Darién, (where health services may not be readily available and where malaria is endemic) showed that treatment with a 10% (70 μg/ml) by volume extract from 15 g of leaves (approximately one blade of the palm) boiled for at least 15 min in 250 ml of water can inhibit the growth of the parasite. We also showed that this extract is not toxic when used at any of the tested concentrations. It is possible that the antimalarial activity is due, in part, to the presence of molecules from the flavonoid family, specifically the epicatechins but, as explained above, most probably in synergy with other compounds. Further studies are required to explore the mechanism of action by which the antimalarial effect is produced and to identify other possible molecules present in the extracts.

Although *in vivo* studies would also be needed to confirm bioavailability, therapeutic dosage or pharmacokinetic parameters, further research could have important health, economic and commercial impacts on remote communities of tropical and subtropical countries where access to medicines is very limited, which grow this tree in abundance and which are precisely those most afflicted by this debilitating and deadly disease.

## Supporting information

S1 Table Crude water-methanol extraction yield for the different parts of the coconut. *Taking water density as a template.
(DOC)

S2 Table *In vitro* antiplasmodial activity of the fractions from the organic extracts of the leaf of *Cocos nucifera* from the Natural Reserve of Punta Patiño. *The samples were tested at 10 μg/ml. The results shown are the average of duplicates.
(DOC)

S1 Fig HPLC profile of the decoction products of *C*. *nuc*ifera leaves. The 15 min decoction of the leaves of the palm tree was analyzed with an HPLC set at 260 nm. A) Fresh aqueous extract. B) Lyophilized extract resuspended in boiling water.
(TIF)
