# Supplementary material for: Analysis of the antiparasitic and anticancer activity of the coconut palm (Cocos nucifera L. ARECACEAE) from the natural reserve of Punta Patiño, Darién
Source: PLoS One. 2019 Apr 2;14(4):e0214193. doi: 10.1371/journal.pone.0214193 (PMC6445518; doi:10.1371/journal.pone.0214193)
Supplement: S1 Table — *Taking water density as a template. (DOC) [file pone.0214193.s001.doc]

**S1 Table. Crude water-methanol extraction yield for the different parts of the coconut**.

| **Source** | **Crude material** | **Organic Extract** | **Yield (%)** |
| --- | --- | --- | --- |
| Leaves | 99.2 g | 32.5 mg | 0.03 |
| Husk | 91.3 g | 88.6 mg | 0.10 |
| Milk | 250 ml | 84.4 mg | 0.03* |
| Meat | 365 g (wet weight) | 22.4 mg | 0.006 |

*Taking water density as a template
