# Supplementary material for: Analysis of the antiparasitic and anticancer activity of the coconut palm (Cocos nucifera L. ARECACEAE) from the natural reserve of Punta Patiño, Darién
Source: PLoS One. 2019 Apr 2;14(4):e0214193. doi: 10.1371/journal.pone.0214193 (PMC6445518; doi:10.1371/journal.pone.0214193)
Supplement: S2 Table — *The samples were tested at 10 μg/ml. The results shown are the average of duplicates. (DOC) [file pone.0214193.s002.doc]

**S2 Table: *In vitro* antiplasmodial activity of the fractions from the organic extracts of the leaf of *Cocos nucifera* from the Natural Reserve of Punta Patiño.**

| **Fraction** | **% Parasitemia Inhibition*** | **SD** |
| --- | --- | --- |
| CPLA | 1.1 | 0.4 |
| CPLB | 0.5 | 0.2 |
| CPLC | 5.0 | 2.3 |
| CPLD | 6.3 | 2.8 |
| CPLE | 0.0 | 0.1 |
| CPLF | 0.0 | 0.4 |
| CPLG | 4.2 | 2.4 |

*The samples were tested at 10 μg/ml. The results shown are the average of duplicates.
